# Selective unresponsiveness to the inhibition of p38 MAPK activation by cAMP helps L929 fibroblastoma cells escape TNF-α-induced cell death

**DOI:** 10.1186/1476-4598-9-6

**Published:** 2010-01-13

**Authors:** Jing Wang, Ruihong Tang, Ming Lv, Jiyan Zhang, Beifen Shen

**Affiliations:** 1 Department of Molecular Immunology, Institute of Basic Medical Sciences, 27 Taiping Road, Beijing 100850, PR China

## Abstract

**Background:**

The cyclic AMP (cAMP) signaling pathway has been reported to either promote or suppress cell death, in a cell context-dependent manner. Our previous study has shown that the induction of dynein light chain (DLC) by cAMP response element-binding protein (CREB) is required for cAMP-mediated inhibition of mitogen-activated protein kinase (MAPK) p38 activation in fibroblasts, which leads to suppression of NF-κB activity and promotion of tumor necrosis factor-α (TNF-α)-induced cell death. However, it remains unknown whether this regulation is also applicable to fibroblastoma cells.

**Methods:**

Intracellular cAMP was determined in L929 fibroblastoma cells after treatment of the cells with various cAMP elevation agents. Effects of cAMP in the presence or absence of the RNA synthesis inhibitor actinomycin D or small interfering RNAs (siRNAs) against CREB on TNF-α-induced cell death in L929 cells were measured by propidium iodide (PI) staining and subsequent flow cytomety. The activation of p38 and c-Jun N-terminal protein kinase (JNK), another member of MAPK superfamily, was analyzed by immunoblotting. JNK selective inhibitor D-JNKi1 and p38 selective inhibitor SB203580 were included to examine the roles of JNK and p38 in this process. The expression of DLC or other mediators of cAMP was analyzed by immunoblotting. After ectopic expression of DLC with a transfection marker GFP, effects of cAMP on TNF-α-induced cell death in GFP+ cells were measured by PI staining and subsequent flow cytomety.

**Results:**

Elevation of cAMP suppressed TNF-α-induced necrotic cell death in L929 fibroblastoma cells via CREB-mediated transcription. The pro-survival role of cAMP was associated with selective unresponsiveness of L929 cells to the inhibition of p38 activation by cAMP, even though cAMP significantly inhibited the activation of JNK under the same conditions. Further exploration revealed that the induction of DLC, the major mediator of p38 inhibition by cAMP, was impaired in L929 cells. Enforced inhibition of p38 activation by using p38 specific inhibitor or ectopic expression of DLC reversed the protection of L929 cells by cAMP from TNF-α-induced cell death.

**Conclusion:**

These data suggest that the lack of a pro-apoptotic pathway in tumor cells leads to a net survival effect of cAMP.

## Background

It is known that persistent stress and depression, which leads to continuously elevated levels of stress hormones such as epinephrine, may increase tumor incidence and promote metastatic growth. Cyclic AMP (cAMP) is the first identified intracellular mediator (second messenger) of hormone action. The downstream effectors of cAMP---protein kinase A (PKA) and cAMP response element-binding protein (CREB)---have been shown to play a role in the tumorigenesis of endocrine tissues [1,2]. Furthermore, it has been long disclosed that cAMP elevation is associated with impaired cell death of various tumor cells [3-10]. Since resistance to cell death has been implicated in cancer pathogenesis, it is of great importance to elucidate the mechanisms by which cAMP plays a pro-survival role in tumor cells.

It is interesting that in non-malignant cells cAMP can either promote or suppress cell death depending on cell type and stimulus used [11-15]. The underlying mechanisms remain the topic of intensive studies. Our recent work has revealed that, at least in fibroblasts, the crosstalk between the cAMP signaling pathway and either JNK (c-Jun N-terminal protein kinase) or p38 pathway plays a key role in the regulation of cell death by cAMP [14,15]. JNK and p38 are members of the mitogen-activated protein kinase (MAPK) superfamily [16-18]. The activation of JNK and p38 are typically mediated by sequential protein phosphorylation through a MAP kinase module, that is, MAPK kinase kinase (MAP3K) → MAPK kinase (MAP2K or MKK) → MAPK, in response to a variety of extracellular stimuli such as UV and tumor necrosis factor alpha (TNF-α) [19-22]. In fibroblasts, the inhibition of JNK by cAMP confers resistance to UV-induced cytotoxicity [15]. cAMP also significantly inhibits TNF-α-induced JNK activation [14]. Even though JNK has been shown to contribute to TNF-α-induced cell death in various types of cells including fibroblasts [23-25], cAMP promotes TNF-α-induced cell death in fibroblasts because it simultaneously inhibits NF-κB activity through dynein light chain (DLC)-mediated suppression of p38 activation [14,15]. Thus, the interplay of the pro-apoptotic pathway(s) and the pro-survival pathway(s) determines the outcome. However, it remains unknown whether the same regulation is also applicable to fibroblastoma cells.

The inhibition of either JNK or p38 by cAMP depends on CREB-mediated transcription and involves upstream MAP2K [14,15]. However, the major effectors of cAMP-mediated inhibition of JNK or p38 activation are different. The induction of DLC is required for cAMP-mediated inhibition of p38 activation [14], whereas the induction of the long form of cellular FLICE-inhibitory protein (c-FLIP_L_) and MAPK phosphatase-1 (MKP-1) is required for cAMP-mediated inhibition of JNK activation [15]. These observations suggest that the inhibition of JNK or p38 by cAMP could be uncoupled in certain cell context. In this work, we report that elevation of intracellular cAMP suppressed TNF-α-induced necrotic cell death in L929 fibroblastoma cells via CREB-mediated transcription. The pro-survival role of cAMP was associated with the lack of an inhibitory effect of cAMP on the pro-survival activation of p38 by TNF-α, even though cAMP significantly inhibited the activation of JNK under the same conditions. The induction of DLC, but not c-FLIP_L _and MKP-1, by cAMP was impaired in L929 cells. p38 selective inhibitor or enforced expression of DLC reversed the protection of L929 cells by cAMP from TNF-α-induced cell death. These data suggest that the lack of a pro-apoptotic pathway in tumor cells leads to a net survival effect of cAMP.

## Materials and methods

### Reagents

Forskolin, prostaglandin E2 (PGE_2_), epinephrine, propidium iodide (PI), and actinomycin D were purchased from Sigma Chemical Co. (St. Louis, MO, USA). Antibodies against phospho-JNK, JNK, phospho-p38, phospho-CREB, CREB, and c-FLIP_L _were from Cell Signaling Technology (Beverly, MA, USA). Antibodies against p38, DLC, actin, and MKP-1 were from Santa Cruz Biotechnology (Santa Cruz, CA, USA). Mouse TNF-α was purchased from R&D Systems (Minneapolis, MN, USA). D-JNKi1 was purchased from BioMol (Plymouth Meeting, PA, USA). SB203580 was from Calbiochem (San Diego, CA, USA). 6-MB-cAMP was from Biolog (Hayward CA, USA). ECL chemiluminescence kit was obtained from Amersham (Arlington Heights, IL, USA).

### Cell culture and transfection

L929 cells were grown in Dulbecco's modified Eagle medium supplemented with 10% fetal bovine serum, 2 mM glutamine, 100 U/ml penicillin, and 100 μg/ml streptomycin. Small interfering RNAs (siRNAs) that target murine CREB were designed based on nucleotides 1084 to 1102 (#1) and 749 to 767 (#2) relative to the translation start site, respectively, and purchased from Dharmacon (Lafayette, CO, USA). pcDNA3.1 Xpress-DLC has been described previously [14]. Transfection was done with Amaxa nucleofection kit V (VCA-1003, program T-20, Gaithersburg, MD, USA), according to the manufacturer's protocol.

### cAMP measurements

Intracellular cAMP was determined in L929 cells using the cAMP enzyme immunoassay kit purchased from Cayman Chemical (Ann Arbor, MI, USA). Samples were prepared exactly as described by the manufacturer.

### Immunoblotting analysis

Immunoblotting analysis was done as previously described [26]. Briefly, adherent cells were washed with PBS and harvested with a cell scraper (Costars, Cambridge, MA, USA) in ice-cold lysis buffer (0.5% NP-40, 20 mM Tris-Cl, pH 7.6, 250 mM NaCl, 3 mM EDTA, 3 mM EGTA, 1 mM sodium orthovanadate, 1 mM DTT, 10 mM PNPP, 10 μg/ml aprotinin). Cell lysates were resolved by SDS-PAGE before transferring to nitrocellulose membranes. Nitrocellulose membranes were then incubated with 5% (w/v) nonfat dry milk in washing buffer (20 mM Tris-Cl, pH 7.6, 150 mM NaCl, and 0.1% Tween 20) for 1 h at 37°C to block nonspecific protein binding. Primary antibodies (1:1000) were diluted in washing buffer containing 3% BSA and applied to the membranes for overnight at 4°C. After extensive washing, the membranes were incubated with peroxidase-conjugated antibodies for 1 h at room temperature and washed again. Immunoreactive bands were visualized with the ECL chemiluminescence kit.

### Cell death assays

Cells were harvested by trypsin digestion. Dual staining with FITC-conjugated Annexin V and PI was carried out to detect the induction of apoptotic cell death. Cells were washed with PBS and resuspended in 200 μL of HEPES buffer (10 mM HEPES, pH 7.4, 150 mM NaCl, 5 mM KCl, 1 mM MgCl_2_, 1.8 mM CaCl_2_) containing 1 μg/ml Annexin V-FITC and 5 μg/ml PI (Annexin V/PI staining kit, BD Biosciences Pharmingen, San Diego, CA, USA). Following incubation for 15 min at room temperature, cells were analyzed by flow cytometry (FACSCalibur; BD Biosciences, Franklin Lakes, NJ, USA). Annexin V-positive/PI-negative cells were apoptotic, whereas Annexin V/PI double positive cells were necrotic. A simple way to detect necrosis is PI staining. After washing with PBS, the pellet was stained with PI at a concentration of 5 μg/ml in PBS and incubated at room temperature in the dark for 5 min, which was followed by flow cytometry.

### Statistical analysis

The data were shown as mean ± standard deviations (SD). The Student's *t*-test was used to compare the difference between the two groups. The difference was considered statistically significant when *p *< 0.05.

## Results

### Forskolin suppresses TNF-α-induced necrotic cell death in L929 fibroblastoma cells

**Figure 1:**
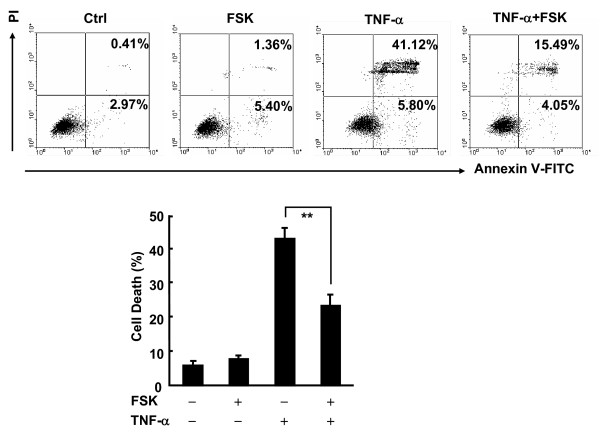
**Forskolin significantly suppresses TNF-α-induced necrotic cell death in L929 fibroblastoma cells**. L929 cells were pretreated with or without forskolin (FSK, 10 μM, 30 min) and then stimulated with 10 ng/ml TNF-α for 24 h or left untreated. Cell death was measured by Annexin-V/PI double staining. The percentages of cell death are shown in the lower panel as mean ± SD; n = 3. Upper panel is representative of three independent experiments. **p < 0.01.

Our previous data have shown that cAMP promotes TNF-α-induced cell death in fibroblasts [14]. However, it remains unknown whether the same regulation is also applicable to fibroblastoma cells. For this purpose, L929 fibroblastoma cells were pretreated with the most widely used cAMP elevation agent forskolin for 30 min [27], followed by stimulation with or without 10 ng/ml TNF-α for 24 h. Surprisingly, cell death assay with Annexin-V/PI double staining revealed that forskolin significantly suppressed TNF-α-induced cell death in L929 fibroblastoma cells (Figure 1). TNF-α induced marginal cell death in fibroblasts [14] (data not shown). However, TNF-α induced massive cell death in L929 cells (Figure 1). The majority of L929 cells undergoing cell death were Annexin-V/PI double positive (Figure 1), consistent with the previous finding that TNF-α treatment of L929 cells leads to a caspase-independent cell death with necrotic phenotype [28,29]. A Simple way to detect necrosis is PI staining [28,29]. Therefore, TNF-α-induced cell death in L929 cells can be simply analyzed with PI staining.

### Various cAMP elevation agents suppress TNF-α-induced cell death in L929 cells

**Figure 2:**
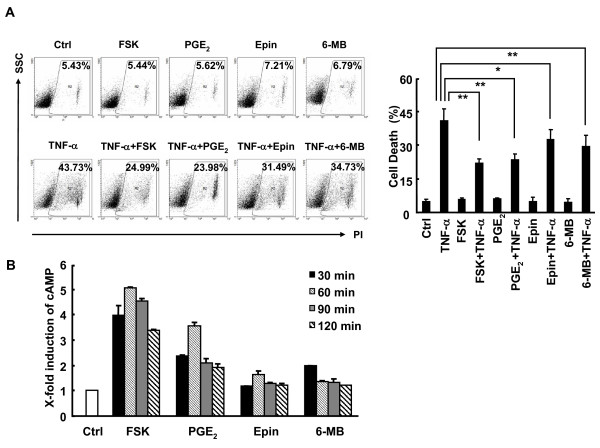
**Various cAMP elevation agents suppress TNF-α-induced cell death in L929 cells**. **A**, L929 cells were pretreated with or without forskolin (10 μM), PGE_2 _(10 μM), epinephrine (Epin, 100 μM), or 6-MB-cAMP (6-MB, 100 μM) for 30 min, followed by stimulation with or without 10 ng/ml TNF-α for 24 h. Cell death was measured by PI staining. The percentages of cell death are shown in the right panel as mean ± SD; n = 3. Left panel is representative of three independent experiments. *p < 0.05. **B**, L929 cells were treated with forskolin (10 μM), PGE_2 _(10 μM), epinephrine (Epin, 100 μM), or 6-MB-cAMP (6-MB, 100 μM) for various times as indicated. Intracellular cAMP was determined using the cAMP enzyme immunoassay kit.

To make sure that forskolin suppresses TNF-α-induced cell death in L929 cells because of the elevation of cAMP, the cells were pretreated with physiologically relevant cAMP inducers PGE_2 _and epinephrine [3,30]. PI staining revealed that under the conditions that pharmacological agent forskolin significantly suppressed TNF-α-induced cell death in L929 cells, physiologically relevant cAMP inducers PGE_2 _and epinephrine exhibited similar effects (Figure 2A). Furthermore, 6-MB-cAMP, a site-selective activator of PKA [14], also suppressed TNF-α-induced cell death in L929 cells (Figure 2A). All these agents led to increased intracellular cAMP in a time-dependent manner (Figure 2B). Moreover, the different ability of these agents to increase the concentration of intracellular cAMP was correlated with the extent these cAMP elevators suppressed TNF-α-induced cell death in L929 cells (Figure 2A). Taken together, these data suggest that cAMP-PKA pathway suppresses TNF-α-induced cell death in L929 cells.

### cAMP suppresses TNF-α-induced cell death in L929 cells via CREB-mediated transcription

**Figure 3:**
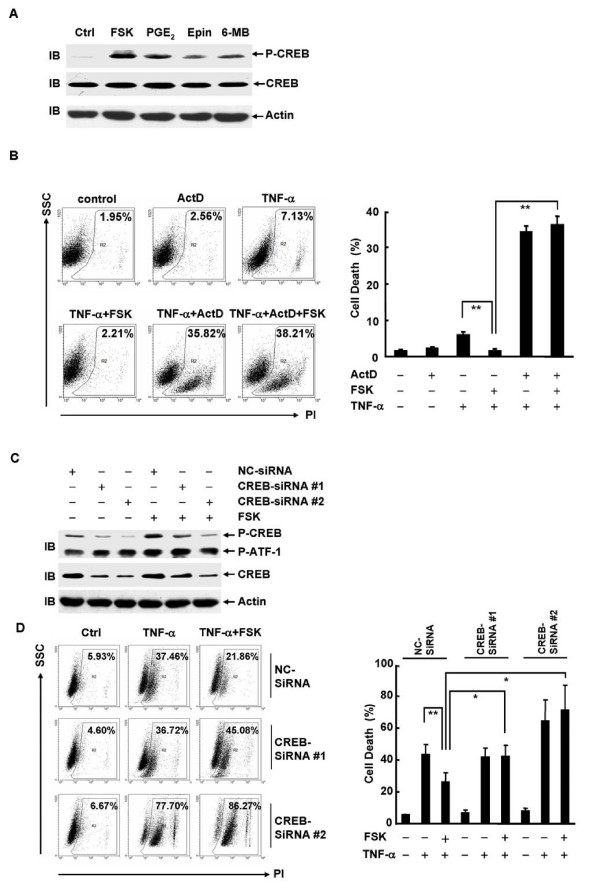
**cAMP suppresses TNF-α-induced cell death in L929 cells via CREB-mediated transcription**. **A**, L929 cells were treated with forskolin (10 μM), PGE_2 _(10 μM), epinephrine (100 μM), or 6-MB-cAMP (100 μM) for 30 min. Phosphorylation of CREB and expression of CREB and actin were analyzed by immunoblotting (IB). **B**, L929 cells were treated with or without actinomycin D (ActD, 1 μg/ml, 30 min) prior to forskolin treatment (10 μM, 30 min), followed by stimulation with or without 2 ng/ml TNF-α for 12 h. Cell death was measured by PI staining. The percentages of cell death are shown in the right panel as mean ± SD; n = 3. Left panel is representative of three independent experiments. **C**, L929 cells were transfected with CREB siRNAs or the negative control (NC) siRNA (200 nM each). After 72 h, cells were stimulated with or without forskolin (10 μM, 30 min). Phosphorylation of CREB and expression of CREB and actin were measured by immunoblotting. **D**, L929 cells were transfected with CREB siRNAs or the negative control siRNA (200 nM each). After 48 h, cells were pretreated with or without forskolin (10 μM, 30 min), followed by stimulation with or without 10 ng/ml TNF-α for 24 h. Cell death was monitored by PI staining. The percentages of cell death are shown in the right panel as mean ± SD; n = 3. Left panel is representative of three independent experiments.

Our previous data suggest that cAMP regulates TNF-α-induced cell death in fibroblasts via CREB-mediated transcription [14]. Consistent with this notion, forskolin-, PGE_2_-, epinephrine-, and 6-MB-cAMP-induced CREB phosphorylation at Ser133 (Figure 3A), which is required for CREB activation [31-33], corresponded to the extent these cAMP elevators activated intracellular cAMP (Figure 2B) and suppressed TNF-α-induced cell death in L929 cells (Figure 2A). To further analyze the mechanism by which cAMP suppresses TNF-α-induced cell death in L929 cells, the cells were pretreated with or without forskolin, followed by treatment with TNF-α in the presence or absence of the RNA synthesis inhibitor actinomycin D. Because 10 ng/ml TNF-α rapidly induced more than 70% cell death in the presence of actinomycin D (data not shown), the concentration of TNF-α was titrated down. 2 ng/ml TNF-α treatment for 12 h led to only 7% cell death in the absence of actinomycin D, which was significantly suppressed by forskolin (Figure 3B). However, in the presence of actinomycin D the same dose of TNF-α resulted in about 35% cell death, though actinomycin D itself showed no detectable effect on the survival of L929 cells (Figure 3B). These data are consistent with the previous finding in the literature that blockade of de novo protein synthesis significantly enhances TNF-α-induced cell death. [34,35] The suppression of TNF-α-induced cell death by forskolin was abolished by actinomycin D (Figure 3B). These data suggest that cAMP suppresses TNF-α-induced cell death in L929 cells in a transcription-dependent manner.

The cAMP pathway activates several transcription factors, including CREB, CREM, and ATF-1 [31-33]. Among them, CREB is the major effector of the cAMP pathway [31-33]. We used CREB siRNAs to test whether CREB mediates the suppression by cAMP of TNF-α-induced cell death in L929 cells. Immunoblotting analysis revealed that CREB siRNAs specifically inhibited CREB expression and abolished the basal and forskolin-stimulated CREB phosphorylation (Figure 3C). Transfection of L929 cells with CREB siRNAs but not the negative control siRNA reversed the suppression of TNF-α-induced cell death by forskolin (Figure 3D). Moreover, CREB siRNA #2, which was more efficient than CREB siRNA #1, led to increased sensitivity of L929 cells to TNF-α-induced cell death. Consistently, the suppression by forskolin of TNF-α-induced cell death was also abolished by ACREB, a specific CREB inhibitor that utilizes its acidic amphipathic extension to prevent the basic region of CREB from binding to DNA [36] (data not shown). Taken together, these data suggest that CREB plays a pro-survival role in TNF-α-induced cell death in L929 cells, and cAMP suppresses TNF-α-induced cell death in L929 cells via CREB-mediated transcription.

### cAMP inhibits TNF-α-induced JNK activation, but not p38 activation, in L929 cells

**Figure 4:**
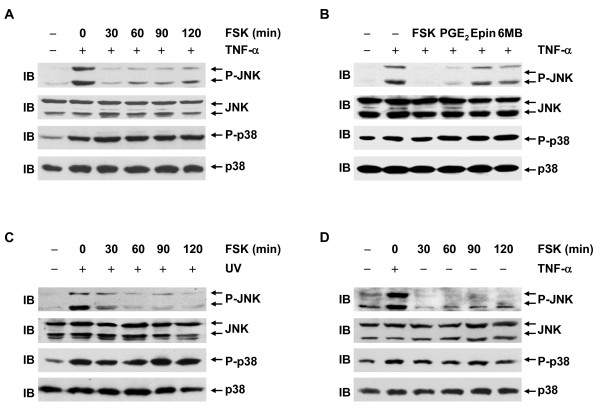
**cAMP inhibits TNF-α-induced JNK activation, but not p38 activation, in L929 cells**. Phosphorylation and expression of JNK and p38 in L929 cells were analyzed by immunoblotting after the following treatment. **A**, L929 cells were pretreated with forskolin (10 μM) for various times as indicated, followed by stimulation with or without 10 ng/ml TNF-α for 15 min. **B**, L929 cells were pretreated with forskolin (10 μM), PGE_2 _(10 μM), epinephrine (100 μM), or 6-MB-cAMP (100 μM) for 30 min, followed by stimulation with or without 10 ng/ml TNF-α for 15 min. **C**, L929 cells were pretreated with forskolin (10 μM) for various times as indicated, followed by stimulation with or without 20 J/m^2 ^UV and incubation for 30 min. **D**, L929 cells were treated with forskolin (10 μM) for various times as indicated or treated with 10 ng/ml TNF-α for 15 min.

Our recent work has revealed that, at least in fibroblasts, the crosstalk between cAMP-PKA-CREB pathway and either JNK or p38 pathway plays a key role in the regulation of cell death by cAMP [14,15]. Now that cAMP suppresses TNF-α-induced cell death in L929 fibroblastoma cells via CREB-mediated transcription, it is of importance to investigate the effects of cAMP on the activation of JNK and p38 in this cell context. For this purpose, L929 cells were pretreated with or without forskolin for various periods of times and then stimulated with TNF-α for 15 min or left untreated. Immunoblotting analysis revealed that TNF-α-induced phosphorylation of JNK at Thr183 and Tyr185, which is required for JNK activation [20], was inhibited by forskolin in a biphasic manner. The inhibition occurred from 30 to 90 min and decreased at 120 min after the pretreatment with forskolin (Figure 4A). The kinetics in the inhibition of JNK activation by forskolin was correlated with its effects on intracellular cAMP (Figure 2B). However, forskolin pretreatment showed no significant effect on TNF-α-induced phosphorylation of p38 at Thr180 and Tyr182, which is required for p38 activation [21,22], under the same conditions (Figure 4A). Similar results were obtained when L929 cells were pretreated with PGE_2_, epinephrine, and 6-MB-cAMP (Figure 4B). The different ability of these agents to inhibit the activation of JNK was correlated with the extent these cAMP elevators increased intracellular cAMP (Figure 2B), activated CREB (Figure 3A), and suppressed TNF-α-induced cell death in L929 cells (Figure 2A). cAMP uncoupled JNK activation and p38 activation not only in response to TNF-α, but also in response to UV (Figure 4C). Furthermore, cAMP inhibited the basal level of JNK phosphorylation, but not p38 phosphorylation, in L929 cells (Figure 4D). Taken together, these data suggest that cAMP uncouples JNK activation and p38 activation in L929 cells.

### JNK activity is required for TNF-α-induced cell death in L929 cells

**Figure 5:**
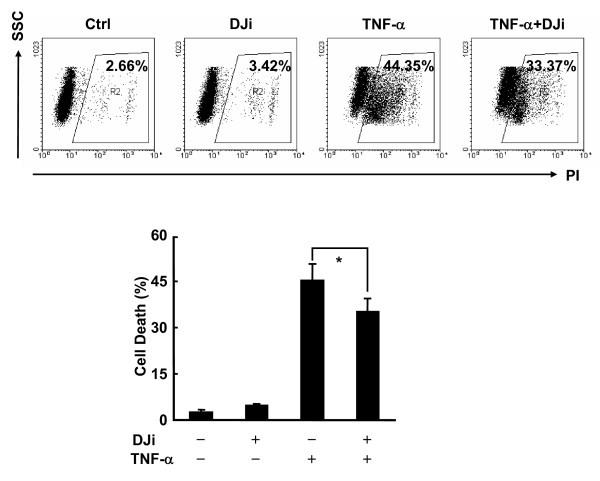
**JNK activity is required for TNF-α-induced cell death in L929 cells**. After pretreatment with or without D-JNKi1 (DJi, 10 μM) for 30 min, L929 cells were treated with or without 10 ng/ml TNF-α for 24 h. Cell death was measured by PI staining. The percentages of cell death are shown in the lower panel as mean ± SD; n = 3. Upper panel is representative of three independent experiments.

Blockade of total JNK activity has been shown to result in impaired cell death in response to TNF-α in various types of cells [23-25]. Because cAMP significantly inhibited TNF-α-induced JNK activation in L929 cells, it is of interest to know the role of JNK in TNF-α-induced cell death in L929 cells. For this purpose, L929 cells were pretreated with or without the selective JNK inhibitor D-JNKi1 (10 μM) [37], and then stimulated with TNF-α or left untreated. Cell death assay revealed that D-JNKi1 significantly suppressed TNF-α-induced cell death in L929 cells (Figure 5), suggesting that JNK contributes to TNF-α-induced cell death in L929 cells. Thus, our data suggest cAMP suppresses TNF-α-induced cell death in L929 cells via, at least partially, inhibition of JNK activity.

### Inhibition of p38 activity reverses the protection of L929 cells by cAMP from TNF-α-induced cell death

**Figure 6:**
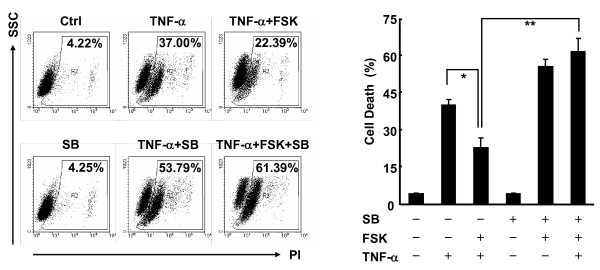
**Inhibition of p38 activity reverses the protection of L929 cells by cAMP from TNF-α-induced cell death**. L929 cells were treated with or without SB203580 (SB, 1 μM, 30 min) prior to forskolin treatment (10 μM, 30 min), followed by stimulation with or without 10 ng/ml TNF-α for 24 h. Cell death was measured by PI staining. The percentages of cell death are shown in the right panel as mean ± SD; n = 3. Left panel is representative of three independent experiments.

Our previous study has shown that cAMP negatively regulates p38 activation, thereby contributing to TNF-α-induced apoptosis in fibroblasts [14]. Now that L929 cells exhibited selective unresponsiveness to p38 inhibition by cAMP and showed impaired cell death in response to TNF-α with cAMP pretreatment, it is possible that the lack of an inhibitory effect of cAMP on the pro-survival activation of p38 by TNF-α helps L929 cells escape TNF-α-induced cell death. To test this scenario, L929 cells were pretreated with or without forskolin, followed by treatment with TNF-α in the presence or absence of the selective p38 inhibitor SB203580 (1 μM) [38,39]. Cell death assay revealed that 1 μM SB203580 significantly reversed the protection of L929 cells by cAMP from TNF-α-induced cell death (Figure 6). Thus, our data suggest that the inhibition of p38 activation over-rides the pro-survival effects of cAMP in TNF-α-induced cell death. Loss of an inhibitory effect of cAMP on p38 activation might help certain types of tumor cells escape from TNF-α-induced cell death.

### Selective unresponsiveness to the inhibition of p38 activation by cAMP results from impaired induction of DLC

**Figure 7:**
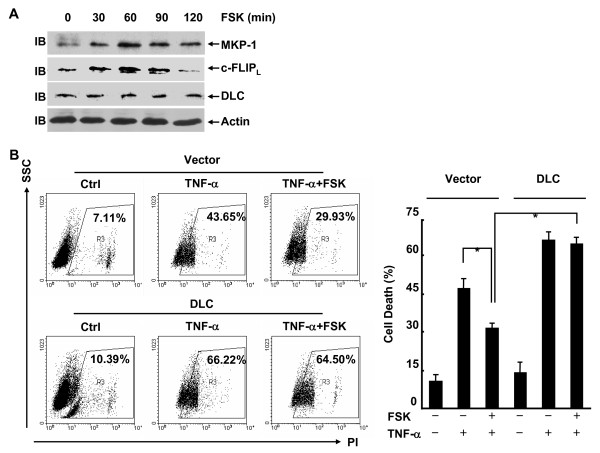
**Selective unresponsiveness to the inhibition of p38 activation by cAMP resulted from impaired induction of DLC**. **A**, L929 cells were treated with forskolin (10 μM) for various times as indicated. The expression of c-FLIP_L_, MKP-1, DLC and actin was analyzed by immunoblotting. **B**, L929 cells were transfected with a mammalian expression vector encoding Xpress-DLC or the empty vector (2 μg each well) with a transfection marker GFP (0.5 μg each well). After 24 h, the cells were pretreated with or without forskolin (10 μM, 30 min) and then stimulated with 10 ng/ml TNF-α for 24 h or left untreated. Cell death was measured by PI staining. The percentages of cell death in GFP+ cells are shown in the right panel as mean ± SD; n = 3. Left panel is representative of three independent experiments.

Our previous studies have revealed that the major effectors of cAMP-mediated inhibition of JNK or p38 activation are different. The induction of DLC is required for cAMP-mediated inhibition of p38 activation [14], whereas the induction of c-FLIP_L _and MKP-1 is required for cAMP-mediated inhibition of JNK activation [15]. Because cAMP uncoupled JNK activation and p38 activation in L929 cells and loss of a cAMP-dependent inhibition of p38 activation might be the key mechanism by which L929 cells escapes TNF-α-induced cell death, it is of great importance to investigate whether DLC induction by cAMP is impaired in L929 cells. For this purpose, L929 cells were treated with forskolin for various periods of times. Immunoblotting analysis revealed that up-regulation of c-FLIP_L _and MKP-1 occurred from 30 to 90 min and decreased at 120 min after the pretreatment with forskolin (Figure 7A), which was correlated with the inhibition of JNK activation (Figure 4). However, there was no detectable up-regulation of DLC under the same conditions (Figure 7A). Thus, DLC induction by cAMP is indeed impaired in L929 cells. Ectopic expression of DLC significantly inhibited p38 activation induced by TNF-α either in the presence or absence of forskolin (data not shown). Furthermore, enforced expression of DLC reversed the protection of L929 cells by forskolin from TNF-α-induced cell death (Figure 7B). Taken together, these data suggest that selective unresponsiveness to the inhibition of p38 activation by cAMP might result from impaired induction of DLC.

## Discussion

It has been long disclosed that cAMP elevation is associated with impaired cell death of various tumor cells [3-10]. In this work, we show that treatment of L929 fibroblastoma cells with various cAMP elevation agents led to increased intracellular cAMP in a time-dependent manner (Figure 2B). cAMP increased following stimulation for 30 and 60 min and thereafter partially declined (Figure 2B). This increase and decline of cAMP were consistent with the so-called "biphasic" inhibition of JNK activation (Figure 4A) and the induction of MKP-1 and c-FLIP_L _(Figure 7A). Elevation of cAMP was associated with suppressed cell death in response to TNF-α (Figure 2A). Even though intracellular cAMP decreased partially after stimulation with forskolin for 90 min, the levels of intracellular cAMP remained much higher than no stimulation control in several hours (Figure 2B and data not shown). Recently, it has been shown that TNF-α induced a gradual, time-dependent increase in cAMP levels that reached a maximum after 8-10 h of stimulation in synovial fibroblasts [40]. Similar increase in cAMP levels were also seen in L929 cells in response to TNF-α [see Additional file 1]. Even though the TNF-α-induced cAMP was weak and showed no statistically significant effect on total intracellular cAMP induced by forskolin [see Additional file 1], it could not be excluded the possibility that the TNF-α-induced cAMP might collaborate with cAMP elevation agents to suppress cell death. Specific blockade of the TNF-α-induced cAMP might address this issue.

Extensive studies have revealed that cAMP might promote the survival of tumor cells by various mechanisms. PKA-mediated phosphorylation of the proapoptotic Bcl-2 family protein BAD at Ser112 sequesters BAD in the cytoplasm through interaction with 14-3-3, thereby preventing BAD interaction with Bcl-2/Bcl-XL on the mitochondrial membrane [3]. Several CREB target genes such as c-FLIP_L_, Bcl-2, and c-IAP-2 have been established to play an anti-apoptotic role [8,9,15,41]. Elevation of cAMP in B cell precursor acute lymphoblastic leukaemia (BCP-ALL) cells is shown to profoundly inhibit DNA damage-induced cell death, which depends on the ability of elevated cAMP levels to quench DNA damage-induced p53 accumulation by increasing the p53 turnover [10].

In this study, our data suggest that cAMP suppresses TNF-α-induced cell death in L929 cells via CREB-mediated transcription (Figure 3 and data not shown). Blockade of transcription with actinomycin D or blockade of CREB activation with CREB siRNAs or ACREB reversed the suppression of TNF-α-induced cell death by cAMP (Figure 3 and data not shown). Therefore, the possible phosphorylation of BAD by PKA is not enough for cAMP to play a pro-survival role in TNF-α-induced cell death in L929 cells. It is not clear how CREB activation mediated the pro-survival effect of cAMP in this cell context. Since the protein levels of Bcl-2 and c-IAP2 have been implicated in the resistance of L929 cells to TNF-α-induced cell death [42,43], the induction of certain anti-apoptotic protein(s) by CREB may play a key role in the suppression by cAMP of TNF-α-induced cell death. Besides directly inhibiting the death machinery, the CREB target gene(s) such as c-FLIP_L _might also suppress TNF-α-induced cell death via regulating JNK activity [15]. The different ability of cAMP elevators to inhibit the activation of JNK (Figure 4B) was correlated with the extent these agents increased intracellular cAMP (Figure 2B), activated CREB (Figure 3A), and suppressed TNF-α-induced cell death in L929 cells (Figure 2A). Moreover, functional inhibition of JNK activity was enough to antagonize TNF-α-induced cell death in L929 cells (Figure 5). Thus, JNK inhibition should be part of the pro-survival cAMP mechanism in TNF-α-induced cell death in L929 cells.

Not only cAMP-stimulated CREB activity protected L929 cells from TNF-α-induced cell death, but also the basal CREB activity might affect the extent of cell death. L929 cells exhibited considerable basal level of phospho-CREB (Figure 3A and 3C). The basal CREB activity might render L929 cells resistant to TNF-α-induced cell death to certain extent by maintaining the protein levels of anti-apoptotic protein(s). The two siRNAs tested reduced CREB levels to different extents (Figure 3C), and the siRNA with greater CREB depletion exhibited a significant effect on TNF-α-induced cell death in the absence of an exogenous cAMP stimulus (Figure 3D). These data suggest that at a certain threshold of CREB depletion, a basal anti-apoptotic effect of CREB is lost, leading to a more significant level of cell death in the presence of TNF-α. This novel finding further suggests a protumorigenic role for CREB.

Despite that cAMP induces a similar activation of CREB in fibroblasts, cAMP promotes TNF-α-induced cell death in fibroblasts because it simultaneously inhibits NF-κB activity through DLC-mediated suppression of p38 activation [21,22]. The inhibitory effect of cAMP on the pro-survival activation of p38 by TNF-α was lacking in L929 fibroblastoma cells, which might be due to loss of a cAMP-dependent induction of DLC. Because the enforced inhibition of p38 activation by using p38 specific inhibitor or ectopic expression of DLC reversed the protection of L929 cells by cAMP from TNF-α-induced cell death, it is the lack of a pro-apoptotic pathway that leads to a net survival effect of cAMP in L929 fibroblastoma cells. It remains unknown why the induction of DLC, but not c-FLIP_L _and MKP-1, by cAMP was impaired in L929 cells. Future studies are required to address this issue.

## Competing interests

The authors declare that they have no competing interests.

## Authors' contributions

JW performed transfection, cAMP measurements, immunoblotting, and cell death assays. RT and ML cultured the cells. JZ designed the study, analyzed the data and wrote the manuscript. BS participated in the design of the study and performed the statistical analysis. All authors read and approved the final manuscript.

## Supplementary Material

Additional file 1 **Effects of TNF-α on the levels of intracellular cAMP with or without forskolin**. L929 cells were treated with 10 ng/ml TNF-α for various periods of time as indicated with or without 10 μM forskolin. Intracellular cAMP was measured using the cAMP enzyme immunoassay kit. Click here for file
