# Supplementary figures and images for: Selective unresponsiveness to the inhibition of p38 MAPK activation by cAMP helps L929 fibroblastoma cells escape TNF-α-induced cell death
Source: Mol Cancer. 2010 Jan 13;9:6. doi: 10.1186/1476-4598-9-6 (PMC2818697; doi:10.1186/1476-4598-9-6)

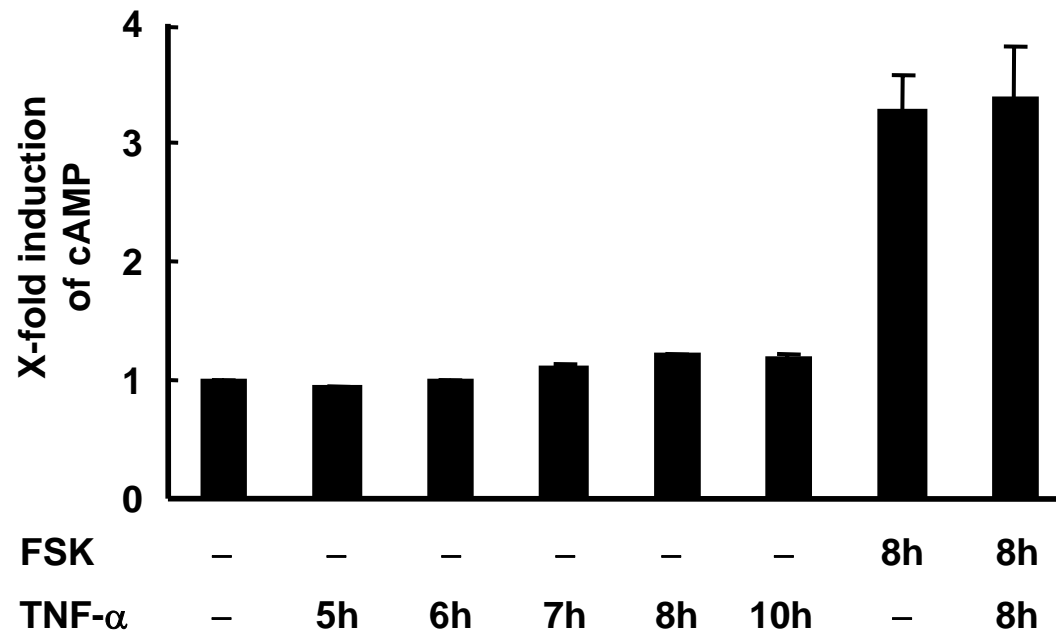

Supplement: Additional file 1 — Effects of TNF-α on the levels of intracellular cAMP with or without forskolin. L929 cells were treated with 10 ng/ml TNF-α for various periods of time as indicated with or without 10 μM forskolin. Intracellular cAMP was measured using the cAMP enzyme immunoassay kit. [file 1476-4598-9-6-S1.PDF]
